# Maternal satisfaction with intrapartum care and associated factors among postpartum women at public hospitals of North Shoa Zone Ethiopia

**DOI:** 10.1371/journal.pone.0260710

**Published:** 2021-12-01

**Authors:** Mulualem Silesh, Tesfanesh Lemma

**Affiliations:** Department of Midwifery, College of Health Science, Debre Berhan University, Debre Berhan, Ethiopia; University of Mississippi Medical Center, UNITED STATES

## Abstract

**Background:**

Maternal satisfaction is an essential indicator of the quality and the efficiency of the health care systems. At a time when efforts are being made globally to reduce maternal and neonatal mortality and morbidity, assessing maternal satisfaction is essential. There is a dearth of studies on maternal satisfaction with intrapartum care, particularly in the study area. This study aimed to assess maternal satisfaction with intrapartum care and associated factors among postpartum women at public hospitals of North Shoa Zone Ethiopia.

**Methods:**

A facility-based cross-sectional study with a systematic random sampling technique was conducted from May1-30/ 2020. Data were entered into EpiData version 4.6 and analyzed using a statistical package for the social sciences version 25. Bivariate and multivariable logistic regression were employed. In multivariable logistic regression analysis, level of statistical significance was declared at variables with p < 0.05 and the strength of the association was measured by an adjusted odds ratio and 95% confidence interval.

**Result:**

Of the total 394 participants, 111 (28.2%) [95% CI: 23.9, 32.5] of postpartum women were satisfied with the intrapartum care. Place of residence [AOR: 1.934; 95% CI (1.183, 3.162)], planned status of the pregnancy [AOR: 2.245; 95% CI, (1.212, 4.158)], number of antenatal care visit [AOR: 2.389; 95% (1.437, 3.974)] and duration of labour [AOR: 2.463; 95% (1.378, 4.402)] were factors significantly associated with maternal satisfaction with intrapartum care.

**Conclusion:**

The proportion of maternal satisfaction with intrapartum care was low. Therefore, designing strategies to enhance maternal satisfaction by strengthening adherence to antenatal care visits, provision of family planning to prevent unplanned pregnancy, and strict utilization of partograph to prevent prolonged labour and childbirth-related complications are crucial.

## Introduction

Providing skilled care during pregnancy, intrapartum, and postpartum periods saves the lives of both mother and newborn [1]. Intrapartum care is care provided to a woman during labour and delivery by midwives and/or other health professionals from the onset of true labour to four hours after the placenta is delivered [2].

Maternal satisfaction is a multidimensional concept that is influenced by the overall health care systems in terms of communication, respect and dignity, emotional support, the physical environment, and quality of care [3–5]; it occurs when maternal expectations are met [6].

Traditionally, the quality of health services was determined by professional practice standards. However, currently, patient perceptions of health care have increasingly become an important indication for determining the quality of care [7]. Mothers’ satisfaction with intrapartum treatment may have both immediate and long-term effects for their health and that of their newborns and it’s essential in maintaining and determining a continuous quality of care on maternal and child health services [8].

As the world health organization (WHO) recommendation, assessing maternal satisfaction during antenatal, intrapartum, and postpartum periods is crucial to improve the quality and efficiency of the healthcare setting [9, 10], in achieving the strategic health plans [11] and universal health coverage by 2030 [12]. High-quality obstetric care in healthcare settings helps to reduce maternal and neonatal morbidity and mortality [13]. The quality of care in developing countries is frequently reported as poor [14]. However, they are still not widely used to improve these initiatives [11].

Globally, there is a target to reduce the maternal mortality ratio (MMR) to less than 70/100, 000 live births and no country should have a MMR greater than 140/100, 000 live births by 2030 [15]. As a WHO report, approximately 810 women died every day in 2017 as a result of pregnancy and childbirth complications; with 94% of them living in low and lower-middle-income countries [1]. Sub-Saharan Africa had a maternal mortality ratio nearly 78 times higher than Australia and New Zealand (542 deaths per 100,000 live births) [16]. In Ethiopia, MMR reported as 401 per 100,000 live births in 2017 [17].

According to the WHO, ensuring patient satisfaction could be a secondary prevention of maternal mortality; satisfied women were more likely to adhere to health care providers’ recommendations, health-seeking behavior and subsequent utilization of the services [18–20] Besides, strengthening institutional delivery with a qualified health professional and provision of high-quality of care to improve maternal and infant health [21], addressing maternal satisfaction is necessary for the efficiency of health services [22].

In developed countries, maternal satisfaction with intrapartum care and its determinants are more closely measured than in developing countries, including Ethiopia [23]. In low and middle-income countries like Ethiopia, where maternal mortality is high and yet skilled birth attendance is low, improving overall satisfaction and quality of care during pregnancy and delivery is a major concern [24]. By 2020, the Ethiopian government planned to increase the proportion of deliveries attended by skilled health personnel from 60 to 90% [25]. However, according to the Ethiopian mini demographic health survey (EMDHS)- 2019, only 48% of women delivered at the health facilities [26].

Although studies on maternal satisfaction have been conducted in Ethiopia, the level of maternal satisfaction with intrapartum care were vary from region to region; in Addis Ababa 19% [2], Gondar 31.3% [27], Hawasa 87.7% [28] and Gamo-Gofa zone 90.2% [29] and there is a dearth of study in the study area.

The findings of this study will provide useful information to health-care providers, hospital managers, local planners and decision makers, and other stakeholders to understand how effectively the services are provided, how well the provider has met the client’s expectations and what changes might be necessary to meet clients’ expectations thereby to increase service utilization which have a substantial favorable impact on mothers’ and newborns’ lives. Moreover, this research hopefully will be used as an input for further studies for other researchers. Therefore, this study aimed to assess maternal satisfaction with intrapartum care and associated factors among postpartum women at public hospitals of North Shoa Zone Ethiopia.

## Methods and materials

### Study setting and period

A facility-based cross-sectional study was conducted at public hospitals of North Shoa Zone, Amhara regional state, Ethiopia. Debre Berhan town is located in the North Shoa Zone about 130 km northeast of the capital city of Ethiopia, Addis Ababa. North Shoa zone is bordered by the Oromia region on the south and the west, on the north by South Wollo, on the northeast by Oromia Zone, and on the east by the Afar region. According to data gathered from the zonal health department, North Shoa Zone is equipped with 10 public hospitals and 97 health centers and on average 3200 mothers gave birth per month at these health facilities; of which 1200 women gave birth at public hospitals. The study was conducted at five public hospitals (Debre Berhan referral hospital, Alem ketema Enat hospital, Ataye hospital, Debre Sina hospital, and Shoa Robit hospital) from May 1–30 / 2020.

### Study population

All selected postpartum women who gave birth at selected public hospitals were the study population.

### Inclusion and exclusion criteria

Postpartum women who gave birth at selected public hospitals during the data collection period were included in the study. Whereas, women who were seriously ill and prepared for referral were excluded.

### Sample size and sampling procedure

The sample size was determined using single population proportion formula by considering the assumptions of the proportion of maternal satisfaction with intrapartum nursing care as 51% from a study done in North Wollo Zone, Ethiopia [30] with a 95% CI (Z@/2 = 1.96), the margin of error 5% and adding 5% of non-response rate. The final sample size was 403. Proportion allocation to population size was employed to determine the numbers of study subjects in each hospital after reviewing the two quarterly institutional delivery reports of the hospitals.

The study subjects were selected by using a systematic random sampling technique and the sampling interval was determined by dividing the number of average monthly delivered mothers by their allocated sample size at each hospital which is; K = N/n ~ 2. Therefore, every 2 postpartum women were included in the study until the required allocated sample at each hospital was achieved.

### Study variables

The dependent variable was maternal satisfaction with intrapartum care and the independent variables were socio-demographic factors (age, place of residence, marital status, educational status, occupational status, and family monthly income) and obstetric and maternal health service-related characteristics (total number of pregnancy, total number of live birth, birth to birth interval, planned status of the pregnancy, history of antenatal care (ANC) follow-up for current pregnancy, number of ANC visit, mode of delivery, current birth outcome, duration of labour and time to be seen by health care provider/s).

### Operational definitions

#### Maternal satisfaction with intrapartum care

Was measured by using 14 items [composed of three subscales; interpersonal care (5 items), information and involvement in the decision making dimension (4 items), and physical birth environment (5 items)] and participants were asked to rate their satisfaction level using a five-point Likert scales (1-strongly disagree to 5-strongly agree). The cut-off point for the overall scale and subscales were determined by the total mean score plus one standard deviation (SD) [31]. Therefore, women who scored ≥ 60 [the total mean score (55.06) + SD (4.94)] were considered as satisfied with the intrapartum care, and those who scored less than 60 were considered as not satisfied.

#### Birth to birth interval

It is the length of time between two successive live births [32].

#### Time to be seen by health care provider

The time elapsed between the arrival of the client to the health facility and the time seen by the healthcare provider/s.

### Data collection procedures and quality control

Data were collected using a structured interviewer-administered questionnaire which was composed of three sections; sociodemographic and economic characteristics, and obstetric and maternal health service-related characteristics, and maternal satisfaction with intrapartum care [30, 31, 33–35] (S1 File). The maternal satisfaction with intrapartum care assessment framework was adopted from women’s satisfaction with hospital-based intrapartum care: a Jordanian study with the Cronbach’s alpha value of 0.88 and 0.76–0.90 for the satisfaction with intrapartum care scale and each sub-scales respectively [31].

The questionnaire was first prepared in English and translated to Amharic (local language) then back to English to ensure consistency. Data collection was carried out in the postpartum unit and confidentiality was kept during the whole process of the study. Five diploma and three bachelors of Science (BSc) degree holder midwives were involved in data collection and supervision respectively. Training for data collectors and supervisors on the data collection procedure and ethical consideration of the study was provided for one day. The questionnaire was pre-tested on 5% of the total sample size among postnatal women at Deneba hospital and necessary amendments were done. The supervisors checked the completeness and consistency of all filled questionnaires daily.

### Data processing and analysis

Data were coded and entered using Epi-Data version 4.6 and then exported to statistical package for the social sciences (SPSS) version 25 for analysis. A descriptive analysis was summarized using proportion, mean, and SDs. Bivariate and multivariable logistic regression analyses were employed to identify factors associated with maternal satisfaction with intrapartum care. Variables observed in a bivariate analysis at p < 0.25 were a candidate for the final model. In multivariable logistic regression analysis, variables at p-value < 0.05 were declared as significantly associated with the outcome variable, and the strength of the association was stated by adjusted odds ratio (AOR) with 95% confidence interval (CI). Finally, the result was summarized and presented in text, tables, and figures.

### Ethical considerations

Ethical clearance to conduct the study was obtained from Jimma University institutional review board (IRB) (Ref. No. IRB000164/20). An official supporting letter was written by the school of nursing and midwifery, Jimma University to the respective health institutions, and formal permission letters were obtained from the administration of the North Shoa Zone health bureau and medical directors of each hospital. Informed written consent was also obtained from each study participant after the objectives of the study were explained. Participation in the study was based on their volunteer status. The information obtained from the participants was kept confidential. Generally, all methods in the study were carried out following the declaration of Helsinki as a statement of ethical principles for medical research involving human subjects.

## Result

### Sociodemographic characteristics of respondents

A total of 394 women were participated in the study; making a response rate of 97.8%. The mean age of the participants was 29.08 (SD ± 4.28). The majority 290 (73.6%) of the participants were found between the age of 25–34 and 198 (50.3%) were rural dwellers. Three hundred fifty-nine (91.1%), 293 (74.4%), and 284 (72.1%) of participants were married, Orthodox Christian, and Amhara in ethnicity respectively. Three hundred thirty (83.8%) and 156 (39.6%) of the study participants were unemployed and had attended primary school respectively. The majority of 271 (68.8%) of the participants’ family monthly income were above 2000 Ethiopian birrs (ETB) (Table 1).

**Table 1:** Socio-demographic characteristics among postpartum women at public hospitals of North Shoa Zone Ethiopia (n = 394).

Characteristics	Category	Frequency(N)	Percent (%)
**Age (in years)**	≤ 24	51	12.9
	25–34	290	73.6
	≥ 35	53	13.5
**Place of residence**	Urban	196	49.7
	Rural	198	50.3
**Religion**	Orthodox	293	74.4
	Muslim	59	15
	Protestant	36	9.1
	Catholic	6	1.5
**Current marital status**	Married	359	91.1
	Not married	35	8.9
**Ethnicity**	Amhara	284	72.1
	Oromo	73	18.5
	Tigrie	21	5.3
	Others^a^	16	4.1
**Educational status**	No formal education	60	15.2
	Primary	156	39.6
	Secondary	122	31.0
	College and above	56	14.2
**Occupational status**	Not employed	330	83.8
	Employed	64	16.2
**Family monthly income (in Ethiopian Birr (ETB))**	≤ 2000	123	31.2
	> 2000	271	68.8

Note:

^**a**^ = Gurage and Argoba.

### Obstetric and maternal health service-related characteristics of respondents

From the total of study participants, 281(71.3%) of women were multipara; 171(60.1%) of them gave birth with a birth interval of ≥ 24 months from their preceding birth. Three hundred sixty-nine (93.7%) of postpartum women had a history of ANC follow-up; among them 200 (54.2%) of women had attended ≥ 4 ANC visits during their current pregnancy. The majority (72.8%) of participants’ current pregnancy was planned and 319 (81%) of women gave birth through vaginal. Regarding the duration of labour, 274 (69.5%) of women gave birth within 12 hours (Table 2).

**Table 2:** Obstetrics and maternal health services characteristics among postpartum women at public hospitals of North Shoa Zone Ethiopia (n = 394).

Characteristics	Category	Frequency(N)	Percent (%)
**Total number of pregnancy (gravidity)**	Primigravida	106	26.9
	Multigravida	288	73.1
**Total number of birth (parity)**	Primipara	113	28.7
	Multi Para	281	71.3
**Birth to birth interval**	<24 months	110	39.1
	≥ 24 months	171	60.9
**Planned status of the pregnancy**	Not planned	107	27.2
	Planned	287	72.8
**History of ANC for current pregnancy**	No	25	6.3
	Yes	369	93.7
**Number of ANC visit (n = 369)**	<4	169	45.8
	≥4	200	54.2
**Mode of delivery**	Vaginal delivery	319	81.0
	Cesarean section	75	19.0
**Current birth outcome**	Alive	360	91.4
	Dead	34	8.6
**Duration of labour**	≤ 12 hrs	274	69.5
	>12 hrs	120	30.5
**Time to be seen by the health care provider/s**	≤ 20 minutes	288	73.1
	>20 minutes	106	26.9

### Maternal satisfaction with intrapartum care

**Fig 1:**
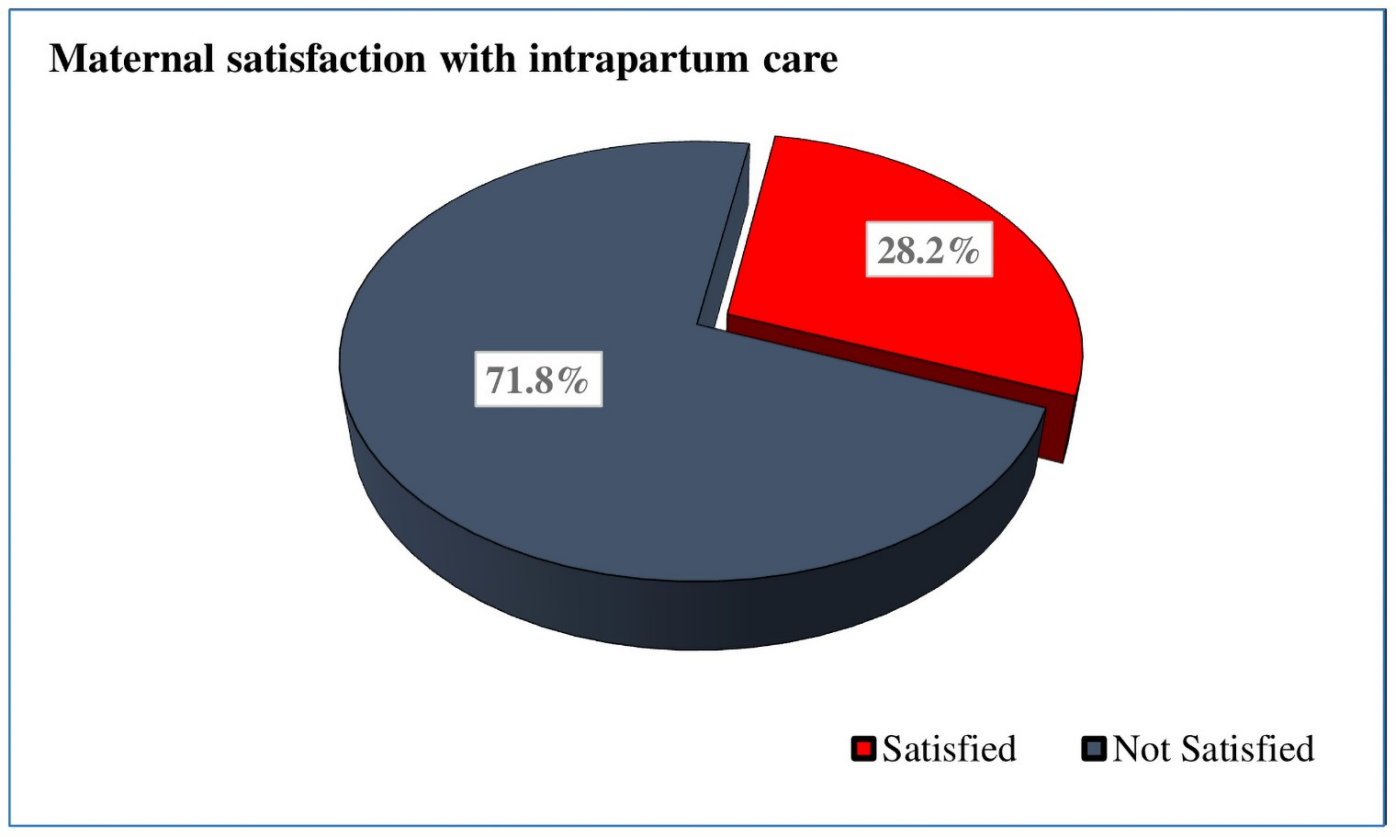
Maternal satisfaction with intrapartum care among postpartum women at public hospitals of North Shoa Zone, Ethiopia (n = 394).

The study found that 111(28.2%) of women were satisfied with the care they received during the intrapartum period (Fig 1). The interpersonal care of the health facilities made 127 (32.2%) participants to be satisfied and 113 (28.7%) women were satisfied with the information they received and the decision-making. One hundred fifty (38.1%) of the respondents were satisfied with the physical birth environment (S1 Table).

### Factors associated with maternal satisfaction with intrapartum care

In bivariate analysis, nine variables (age, place of residence, planned status of the pregnancy, number of ANC visits, current birth outcome, mode of delivery, time to seen by health care provider, marital status, and duration of labour) were a candidate to multivariable logistic regression analysis. In multivariable logistic regression analysis, place of residence, planned status of the pregnancy, number of ANC visits, and duration of labour were significantly associated with maternal satisfaction with intrapartum care.

Mothers from rural residence were almost two times more likely satisfied with the overall intrapartum care provided compared to mothers from urban [AOR: 1.934; 95% CI (1.183, 3.162)]. Mothers who reported their current pregnancy was planned were 2 times more likely satisfied with the intrapartum care compared to their counterparts [AOR: 2.245; 95% CI, (1.212,4.158)]. Mothers who had attended ANC visit four and above in their current pregnancy were 2.4 times more likely to be satisfied with intrapartum care compared to women who had attended ANC less than four visits [AOR: 2.389; 95% (1.437, 3.974)]. The likelihood of maternal satisfaction with the intrapartum care was 2.4 times higher among mothers’ labour persists ≤ 12 hours than mothers stayed in labour more than 12 hours [AOR: 2.463; 95% (1.378, 4.402)] **(**Table 3).

**Table 3:** Factors associated with maternal satisfaction with intrapartum care among postpartum women at public hospitals of North Shoa Zone Ethiopia (n = 394).

Variable	Satisfaction		P-Value	COR, 95%CI	AOR, 95%CI
	Not Satisfied	Satisfied			
**Age (in years)**					
≤ 24	35(68.6%)	16(31.4%)	0.363	0.818(0.362,1.849)	0.646(0.252,1.657)
25–34	214(73.8%)	76(26.2%)	0.073	0.636(0.342,1.181)	0.526(0.261,1.062)
≥ 35	34(64.2%)	19(35.8%)		1	1
**Place of residence**					
Urban	153(78.1%)	43(21.9%)			1
Rural	130(65.7%)	68(34.3%)	0.009	1.86(1.189, 2.912)	1.934(1.183, 3.162)**
**Marital status**					
Not married	22(62.9%)	13(37.1%)	0.892	1.574(0.763,3.246)	1.064(0.435,2.601)
Married	261(72.7%)	98(27.3%)		1	1
**Planned status of the pregnancy**					
Not planned	91(85%)	16(15%)		1	
Planned	192(66.9%)	95(33.1%)	0.010	2.814(1.567,5.054)	2.245(1.212,4.158)**
**Mode of delivery**					
Vaginal	223(69.9%)	96(30.1%)		1	1
Cesarean section	60(80%)	15(20%)	0.226	0.581(0.314,1.073)	0.659(0.336,1.295)
**Current birth outcome**					
Alive	252(70%)	108(30%)		1	1
Dead	31(91.2%)	3(8.8%)	0.059	0.226(0.068, 0.75)	0.298(0.085,1.048)
**Number of Antenatal Care visits (n = 369)**					
< 4 visit	136(80.5%)	33(19.5%)		1	1
≥ 4 visit	131(65.5%)	69(34.5%)	0.001	2.171(1.344, 3.51)	2.389(1.437,3.974)**
**Time to seen by the health care provider/s**					
≤ 20 minutes	201(69.8%)	87(30.2%)	0.059	1.479(0.879,2.487)	1.720(0.98,3.018)
>20 minutes	82(77.4%)	24(22.6%)		1	1
**Duration of labour**					
≤ 12 hours	185(67.5%)	89(32.5%)	0.002	2.143(1.265,3.630)	2.463(1.378,4.402)**
>12hours	98(71.8%)	22(18.3%)		1	1

Note:

** *=* Variables significant at P < 0.05 ***1*** *= reference category*.

## Discussion

Providing institutional delivery with enhanced maternal satisfaction is one of the most effective techniques for reducing maternal mortality rates in developing countries like Ethiopia [27]. Measuring maternal satisfaction with intrapartum care help to assess women’s experiences with intrapartum care, the quality of care they received, and identify what changes to be required [2]. This study revealed that, the overall prevalence of maternal satisfaction with intrapartum care was 28.2% and place of the residence, planned status of the pregnancy, number of ANC visit and duration of labour were factors associated with maternal satisfaction with intrapartum care.

This study showed that the overall maternal satisfaction with intrapartum care was found to be 111 (28.2%) [95% CI: 23.9, 32.5] which was consistent with previous studies conducted in Gondar, Ethiopia (25.3–31.3%) [27, 36]. However, this finding was higher than studies done in Jordan (17.3%) [31], Eritrea (20.8%) [37], and Addis Ababa, Ethiopia (19%) [2]. This discrepancy might be due to the difference in time of the study conducted, level of health facilities and sociodemographic characteristics of participants; media exposure, and clients’ expectation about availability and quality of services at the health facilities. The odds of maternal satisfaction with delivery services were lower among women who reside in rural area and had no formal education [28, 34]. On the other hand, it was lower than studies conducted in Mid-Western Nepal (89.88%) [38], Iraq (40.3%) [39], Saudi Arabia (89.5%) [40], Mozambique (92.5%) [4], Kenya (54.5%) [41], and Ethiopia (47.6% - 88%) [33–35, 42–46]. The possible variation might be due to the difference in quality of service provided at health facilities and satisfaction measurement tool used.

Mothers from rural area were almost two times more likely to be satisfied with intrapartum care compared to mothers from the urban residence. This finding was consistent with previous studies conducted in the Bench-Maji zone [34] and Addis Ababa [47]. However, it is contrary to the study done in Gondar [27]. This could be due to the difference in sociodemographic characteristics of the study population; women who reside in rural area more likely to have lower expectations regarding to quality of service provided and less exposure to the healthcare delivery system than women from urban residence. The study done in Gondar showed that, 17.3% of participants were living in rural area. Whereas, in this study 50.3% of the participants were residing in rural area [27].

Planned status of current pregnancy was a significant predictor of maternal satisfaction with intrapartum care; women who had planned pregnancy were two times more likely to be satisfied with intrapartum care than those who did not plan. This finding is similar with the studies conducted in Nairobi, Kenya [48], Adama, Ethiopia [35], Jimma, Ethiopia [43], and Amhara, Ethiopia [36]. This could be due to psychological and financial readiness for the impending arrival of their child. Also, women with unintended pregnancy were less likely to receive ANC compared to women with intended pregnancy [49]. This will eventually have a negative impact on the clients’ awareness towards health-care services and familiarity with health facilities prior to delivery service.

In addition this study revealed that, frequent ANC had a significant impact on maternal satisfaction with intrapartum care. The odds of maternal satisfaction with intrapartum care was two times higher among women who had four and above ANC visits compared to those women who had less than four visits. A similar finding was presented by another studies conducted in Ethiopia [29, 33, 34]. Women who had ANC follow-ups were two times more likely to be satisfied with intrapartum care as compared to women who had no ANC follow-up [43]. This could be related to women’s familiarity with health facilities prior to delivery service which allows them to have reasonable expectations.

The other predictor of maternal satisfaction with intrapartum care was duration of labour. Maternal satisfaction was 2.4 times higher among mothers whose labour lasted ≤ 12 hours than women whose labour lasted more than 12 hours. This finding was in line with studies conducted in Nekemte [44], Debre-Markos [45], Wolaita zone [18], and Wolaita-Sodo [33]. This could be due to suffering from labor pain and childbirth-related complication less likely if the women stayed in labour for short duration.

Furthermore, the finding of this study implies that majority of labouring mothers were not satisfied with the care they received during intrapartum period. This would have a negative impact on the subsequent utilization of health services if clients’ expectations is not enhanced. This study could help for policymakers and implementers as an input to design strategic policies and interventions to enhance maternal satisfaction in the health-care system.

### Limitation of the study

The cross-sectional nature of the study does not show a cause and effect relationship, limited to public hospitals, and potential response biases related to social desirability were the limitations of this study. However, authors attempted to reduce biases by interviewing while the women prepared for discharge from the health facilities.

## Conclusion

In general, the study found that the overall maternal satisfaction with intrapartum care was low. Participants’ place of residence, planned status of the pregnancy, number of antenatal care visits, and duration of labour were factors significantly associated with maternal satisfaction with intrapartum care.

Satisfaction with the available services was a key factor affecting the health-seeking behavior and service utilization. Considering this, policymakers, health professionals, zonal health service planners, and hospital administrators need to design strategies to enhance maternal satisfaction by strengthening the adherence to ANC visits, the provision of family planning to prevent unplanned pregnancy and strict utilization of partograph to monitor progress of labour and prevent prolonged labour are essential. Further large population-based studies on the determinants of maternal satisfaction with intrapartum care should be considered to address the cause and effect relationship.

## Supporting information

S1 File Questionnaire. (PDF)

S1 Table Frequency distribution of maternal satisfaction with total scale and subscales. (PDF)

## Abbreviations

ANC: Antenatal care
AOR: Adjusted odds ratio
CI: Confidence interval
COR: Crude odds ratio
EMDHS: Ethiopian Mini demographic health survey
MMR: Maternal mortality ratio
SD: Standard deviation
WHO: World Health Organization
