# Supplementary material for: Maternal satisfaction with intrapartum care and associated factors among postpartum women at public hospitals of North Shoa Zone Ethiopia
Source: PLoS One. 2021 Dec 1;16(12):e0260710. doi: 10.1371/journal.pone.0260710 (PMC8635333; doi:10.1371/journal.pone.0260710)
Supplement: S1 File — (PDF) [file pone.0260710.s001.pdf]

Maternal satisfaction with intrapartum care and associated factors among women who gave birth at public hospitals of north Shoa Zone, Ethiopia

Authors

Mulualem Silesh [1*] and Tesfanesh Lemma [1]

[1] Department of Midwifery, College of Health Science and Medicine, Debre Berhan University, Debre Berhan, Ethiopia.

*Corresponding author: *sileshmulualem22@gmail.com* (MS)

Questionnaire (English version)

S.N.	Question	Possible Response	Skip pattern
**PART 1: Socio-demographic and Economic characteristics**			
101	Age	__________ (in years)	
102	Residence	1. Urban<br>2. Rural	
103	Current marital status	1. Single<br>2. Married<br>3. Widowed<br>4. Divorced	
104	Religion	1. Orthodox<br>2. Protestant<br>3. Muslim<br>4. catholic<br>5. Other (specify)________	
105	Educational status of the woman	1. No formal education<br>2. Primary education<br>3. Secondary education<br>4. College and above	
106	Ethnicity	1. Amhara<br>2. Oromo<br>3. Tigre<br>4. Gurage<br>5. Other(specify)________	
107	Occupational status	1. Unemployed<br>2. Employed	
108	Family's monthly income (in Ethiopian birr)	___________ Birr	
**PART 2: Obstetric and maternal health service characteristics**			
201	Total number of pregnancy (include live birth, abortion and still birth)	_________(in number)	
202	Total number of birth (include live birth, and still birth)	_________(in number)	If 1, skip to 204

203	Birth to birth interval from the index child?	________(in months)	
204	Present birth planned?	1. Yes 2. No	
205	Did you have ANC follow up during the current pregnancy?	1. Yes<br>2. No	If "2", skip to 207
206	If yes, How many times you had attend ANC?	________(number of visit)	
207	Mode of delivery of current birth?	1. vaginal delivery<br>2. Cesarean section<br>3. Other (specify)	
208	Current birth outcome?	1. Alive<br>2. Dead	
209	How long the duration of labour persists?	___________(in hours)	
210	Time to be seen by the health care providers	___________(in months)	

PART 3: Satisfaction with intrapartum care

S.N	Questions	Responses	Possible choices
	**Interpersonal care**		1. Strongly disagree<br>2. disagree<br>3. Neutral<br>4. Agree<br>5. Strongly agree
301	Health care providers were friendly and welcoming when you arrived at the hospital		
302	Health care providers were encouraging and reassuring during labour and/or birth.		
303	Health care providers were helpful during labour and/or birth.		
304	During labour and/or birth physicians were more helpful.		
305	The overall care during labour and birth was good		
	**Information provision &decision making**		

306	The health care provider always kept you informed about what was happening during labour &/ or birth.		
307	During labour and/or birth decisions made without taking your wishes into account		
308	You felt pressured to have the baby quickly		
309	You felt labour was taken over by strangers and/or machines		
	**Physical birth environment**		
310	During labour&/or birth the level of light was adequate		
311	During labour&/or birth the room was spacious and adequate for your need		
312	During labour&/or birth the level of noise was appropriate		
313	During labour&/or birth trays and other equipment's were clean		
314	During labour&/or birth you were able to find the supplies that you needed		

Thank you for your participation
