# Supplementary material for: Maternal satisfaction with intrapartum care and associated factors among postpartum women at public hospitals of North Shoa Zone Ethiopia
Source: PLoS One. 2021 Dec 1;16(12):e0260710. doi: 10.1371/journal.pone.0260710 (PMC8635333; doi:10.1371/journal.pone.0260710)
Supplement: S1 Table — (PDF) [file pone.0260710.s002.pdf]

S1 Table: Frequency distribution of maternal satisfaction with intrapartum care scale and subscale among postpartum women at public hospitals of North Shoa Zone Ethiopia (n = 394)

Characteristics	Category	Mean	SD	Maternal Satisfaction	
				Satisfied	Not satisfied
**Total Scale**	Maternal satisfaction scale (14 items)	55.06	4.96	111(28.2%)	283(71.8%)
**Subscale 1**	Interpersonal care (5 items)	20.13	2.05	127(32.2%)	267(67.8%)
**Subscale 2**	Information and decision making (4 items)	15.05	2.4	113(28.7%)	281(71.3%)
**Subscale 3**	Physical birth environment (5 items)	19.88	1.94	150(38.1%)	244(61.9%)
